# ReHoGCNES-MDA: prediction of miRNA-disease associations using homogenous graph convolutional networks based on regular graph with random edge sampler

**DOI:** 10.1093/bib/bbae103

**Published:** 2024-03-19

**Authors:** Yufang Zhang, Yanyi Chu, Shenggeng Lin, Yi Xiong, Dong-Qing Wei

**Affiliations:** School of Mathematical Sciences and SJTU-Yale Joint Center for Biostatistics and Data Science, Shanghai Jiao Tong University, Shanghai 200240, China; Peng Cheng Laboratory, Shenzhen, Guangdong 518055, China; Zhongjing Research and Industrialization Institute of Chinese Medicine, Zhongguancun Scientific Park, Meixi, Nanyang, Henan, 473006, China; Department of Pathology, Stanford University School of Medicine, Stanford, CA, 94305, USA; State Key Laboratory of Microbial Metabolism, School of Life Sciences and Biotechnology, and Joint Laboratory of International Cooperation in Metabolic and Developmental Sciences, Ministry of Education, Shanghai Jiao Tong University, Shanghai 200240, China; State Key Laboratory of Microbial Metabolism, School of Life Sciences and Biotechnology, and Joint Laboratory of International Cooperation in Metabolic and Developmental Sciences, Ministry of Education, Shanghai Jiao Tong University, Shanghai 200240, China; Shanghai Artificial Intelligence Laboratory, Shanghai, 200232, China; Peng Cheng Laboratory, Shenzhen, Guangdong 518055, China; Zhongjing Research and Industrialization Institute of Chinese Medicine, Zhongguancun Scientific Park, Meixi, Nanyang, Henan, 473006, China; State Key Laboratory of Microbial Metabolism, School of Life Sciences and Biotechnology, and Joint Laboratory of International Cooperation in Metabolic and Developmental Sciences, Ministry of Education, Shanghai Jiao Tong University, Shanghai 200240, China

**Keywords:** miRNA-disease associations, graph convolutional network, regular graph, graph sampler

## Abstract

Numerous investigations increasingly indicate the significance of microRNA (miRNA) in human diseases. Hence, unearthing associations between miRNA and diseases can contribute to precise diagnosis and efficacious remediation of medical conditions. The detection of miRNA-disease linkages via computational techniques utilizing biological information has emerged as a cost-effective and highly efficient approach. Here, we introduced a computational framework named ReHoGCNES, designed for prospective miRNA-disease association prediction (ReHoGCNES-MDA). This method constructs homogenous graph convolutional network with regular graph structure (ReHoGCN) encompassing disease similarity network, miRNA similarity network and known MDA network and then was tested on four experimental tasks. A random edge sampler strategy was utilized to expedite processes and diminish training complexity. Experimental results demonstrate that the proposed ReHoGCNES-MDA method outperforms both homogenous graph convolutional network and heterogeneous graph convolutional network with non-regular graph structure in all four tasks, which implicitly reveals steadily degree distribution of a graph does play an important role in enhancement of model performance. Besides, ReHoGCNES-MDA is superior to several machine learning algorithms and state-of-the-art methods on the MDA prediction. Furthermore, three case studies were conducted to further demonstrate the predictive ability of ReHoGCNES. Consequently, 93.3% (breast neoplasms), 90% (prostate neoplasms) and 93.3% (prostate neoplasms) of the top 30 forecasted miRNAs were validated by public databases. Hence, ReHoGCNES-MDA might serve as a dependable and beneficial model for predicting possible MDAs.

## INTRODUCTION

MicroRNA (MiRNA), a category of minuscule RNA comprising ~22 nucleotides and classified as noncoding RNA, was initially unearthed in 1993 [1, 2], which significantly influences the modulation of target gene expression through inducing cleavage degradation, halting translation and other structural regulatory mechanisms [3]. A growing number of research highlights the involvement of miRNAs in crucial biological functions including cell growth and diversification, senescence-induced apoptosis, immune reactions, signaling pathways, tumor penetration and viral incursions [4–8]. For example, a simultaneous decline in mir-103 or mir-107 levels alongside an elevation in silk protein levels was observed in a transgenic mouse model replicating Alzheimer’s disease [9]. Gao *et al.* [10] identified the early onset dysregulation of mir-145 and mir-199 expression in the early stages of hepatitis B virus-associated multistep liver cancer. A recent study has shown that mir-23, mir-24 and mir-27 contain harbor potential therapeutic elements for tackling ischemic cardiac and vascular disorders [11].

Hence, unveiling potential miRNA-disease connections could furnish a more profound comprehension of the molecular underpinnings of diseases, enabling early intervention. Efficient harnessing of information regarding miRNA-disease relations can enhance the diagnosis, prognosis and remediation of intricate human diseases [12, 13]. As of now, the majority of the miRNA-disease associations (MDAs) are sourced from biological experiments, which are both time-intensive and costly [14]. Instead, computational methods are developed to predict potential MDAs to help researchers prioritize the potential MDAs for experimental validation [15, 16]. Generally, these computational methods are categorized into four main types: similarity-based methods [17–23], traditional machine learning [24–28], deep learning [29–31] and graph neural network (GNN) algorithms [32–34]. Table S1 shows summary of four computational methods mentioned above for MDA prediction. For example, DNRLMF-MDA proposed by Yan *et al*. [23] computes the probability that a miRNA would interact with a disease by a logistic matrix factorization method, where latent vectors of miRNAs and diseases represent the properties of miRNAs and diseases, respectively, and further improve prediction performance via dynamic neighborhood regularized. Xuan *et al*. [24] developed a model, termed HDMP, grounded on methods of similarity. The functional resemblance among miRNAs was deduced based on disease terminologies and the similarity of disease phenotypes. Nonetheless, HDMP falls short in predicting prospective miRNAs for novel diseases devoid of any known associated miRNAs. A variety of traditional machine learning- and deep learning-based frameworks have been devised to predict possible MDAs. Chen *et al*. [35] introduced RKNNMDA, which employs SVMs to rank K-nearest neighbors (KNN) and uses weighted voting to predict MDAs. Zhao *et al.* [27] employed adaptive boosting for MDA prediction, utilizing a decision tree as a basic classifier before amalgamating weak classifiers to compose a robust classifier based on respective weights. These algorithms were leveraged to derive effective feature representations and tackle the specific optimization challenge to predict reliable MDAs. Deep learning approaches have surpassed traditional machine learning methods in terms of accuracy [36, 37]. A common suggestion to augment accuracy via deep learning is to employ more data for training, a strategy that typically doesn’t augment classical machine learning algorithms’ accuracy, necessitating researchers to seek more refined methods for accuracy enhancement. Wang *et al.* [29] present a novel data-driven end-to-end learning-based method of neural multiple-category miRNA-disease association prediction (NMCMDA) for predicting multiple-category MDAs. As deep learning is a black box model, hyperparameters selection and network design are challenging [38, 39]. Moreover, deep learning methods require significant execution time with the substantial compute and memory operations [40].

GNNs, especially graph convolutional networks (GCNs), are widely used in bioinformatics applications due to their robust data comprehension and cognitive capabilities. They are employed in various tasks such as drug-target interactions predictions [41–45], gene-disease association identification [46–48], drug–drug interaction predictions [49–52] and so on. For example, the GraRep technique, as introduced by [55], integrates principles from similarity-based, machine learning and GNN methodologies together. It builds a heterogeneous GCN encompassing miRNA, disease, drug, protein, lncRNA, along with their interactions. Additionally, disease similarity data are taken into account when forming embedding representations. In the final stage, the random forest (RF) algorithm is employed to predict potential MDAs. GNNs have strong data and knowledge representation capabilities, which can not only express the independent characteristics of samples (nodes), but also express the connections (links) between samples of the same type or even different types. Unlike the pictures and sequences that are data in Euclidean space and their graph structure is fixed, GNNs deal with data like biological data in non-Euclidean spaces with extremely flexible graph structures. Several studies have endeavored to employ varied GCN architectures for predicting MDAs, and these can be broadly categorized into three classes: (i) pairwise GCNs [53–55], which deploy two separate GCNs to derive embeddings for miRNAs and diseases, thereafter predicting MDAs. However, this graph structure overlooks the relationships between miRNA-disease pairs (MDPs). (ii) Link prediction on a bipartite graph [56], where both miRNAs and diseases are regarded as nodes while MDAs are regarded as edges. In this setup, a large number of negative samples are used as edges during node updates, leading to the over-smoothing issue due to the inclusion of numerous false neighbors. (iii) Node prediction on a fully connected graph [57–59], where the graph’s high density causes the embeddings of each node to converge toward uniformity as nodes continue to update, thereby also triggering the over-smoothing problem.

**Figure 1:**
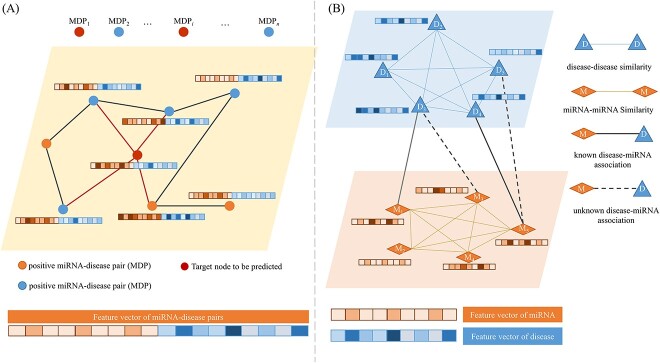
Basic structures of homogenous graph (A) and heterogeneous graph (B) used in MDA prediction task.

A homogenous graph refers to a graph where all nodes and edges belong to the same type while in a heterogeneous graph, nodes and edges may have multiple types, representing different entities or relationships. The classification of graphs into homogenous and heterogeneous is based on the properties of nodes and edges. Figure 1 illustrated the basic structures of homogenous graph (A) and heterogeneous graph (B) used in MDA task. Path-based MiRNA-disease association (PBMDA) prediction model was proposed by You [85] constructed a heterogeneous graph consisting of three interlinked sub-graphs and further adopted depth-first search algorithm to infer potential MDAs. Chen [17] designed a Laplacian score of homogenous graphs to calculate the global similarity of networks and proposed a global similarity method based on a two-tier random walk to reveal the correlation between miRNAs and diseases.

Based on the different distributions of node degrees, graphs can be classified into regular graph and non-regular graph. In a regular graph, each node has the same degree, meaning that every node has the same number of neighbors while nodes in a non-regular graph may have different degrees, meaning that nodes in the graph can have different numbers of neighbors. Table 1 summarized different graph structures and prediction models for MDA prediction. Regular undirected graph, which is a simple type of graph structures but is hard to generate, not only has good combinatorial properties but also has strong algebraic constraints, which haven’t been widely used in bioinformatics area [68, 69]. Fully connected network based on similarity is a common method to construct regular graphs. NIMCGCN [54] first learn miRNA and disease latent feature representations from fully connected homogenous miRNA and disease similarity graph, respectively. Then, learned features were input into a novel neural inductive matrix completion model to generate an association matrix completion. Another common method is K-NN method to construct regular graphs. ProteinGCN [76] represented a protein as a graph in which atoms are represented by nodes, and edges connect to $k$ nearest neighbors of each node atom. This representation has rotation invariance and reduces the information redundancy brought about by fully connected networks. Chu *et al.* [32] designed a homogenous graph of MDPs and the edge is constructed between the node and its $k$ nearest neighbors based on the node information. Moreover, the aforementioned methods only accomplish a portion of the MDAs predictions, neglecting the predictive task for new diseases, new miRNAs and their associations. Hence, it’s worthwhile to investigate new GCN architectures to fully harness the structural attributes of the biological graph. Additionally, validation or independent testing should be conducted to evaluate the overall predictive efficacy on new miRNAs and diseases that were not included in the training dataset.

**Table 1:** Summary of different graph structures and prediction types of MDA

Algorithm	Graph structure				Supervised model		Others
	Homogenous graph	Heterogeneous graph	Regular graph	Non-regular graph	Node prediction	Link prediction	
MDA-GCNFTG [32]	√		√		√		
VGAE-MDA [53]		√		√			√
LAGCN [78]		√		√		√	
DEJKMDR [79]		√		√		√	
NSAMDA [80]		√		√		√	
HGANMDA [81]		√		√		√	
HLGNN-MDA [82]	√			√	√		
PBMDA [85]		√		√			√
GSTRW [17]	√		√				√
NIMCGCN [54]	√		√				√
ReHoGCN^a^	√		√		√		
UReHoGCN^a^	√			√	√		
HeGCN^a^		√		√		√	

^a^Model proposed in our work.

Despite numerous efforts [60–62] to minimize training expenditures with GCNs, these approaches still encounter hurdles in terms of accuracy, scalability and training complexity . The ‘neighbor explosion’ phenomenon is a common hurdle when dealing with complex large graphs leading to increased complexity of node representation and stochastic gradient calculation will exponentially increase with the increasing number of message passing layers. Additionally, stacking multiple layers of GCN often results in over-smoothing or overfitting issues, causing nodes tend to have similar representations after aggregation operations as the neural network goes deeper. Researchers have proposed various graph sampling techniques to reduce the number of nodes involved in message passing, thereby lowering training costs. The most common techniques include node sampling (such as GraphSAGE [63], PinSage [64], VRGCN [65]), layer sampling (such as FastGCN [66], ASGCN [67]) and edge sampling.

In light of these challenges, this study introduces a novel ReHoGCNES-MDA method, grounded on a regular undirected graph, for MDA prediction, and evaluates it across four distinct prediction tasks, incorporating both aforementioned viewpoints simultaneously. Initially, we proposed a homogenous GCN with a regular graph structure (ReHoGCN) utilizing $k$-nearest neighbors (k-NN) algorithms [70] to efficiently and adequately probe information like node features (i.e. miRNAs and diseases) and network topology (i.e. miRNA-disease links). For comparison, we also introduced homogenous GCN non-regular graph structure (UReHoGCN) via $k$-means [71] algorithms, and heterogeneous GCN (HeGCN). A random edge sampler (ES) strategy was utilized to hasten processes and diminish training complexity. Subsequently, these three structurally distinct GCNs were tested on four experimental tasks concerning MDA predictions for the first time: specifically, predicting new associations between known miRNAs and known diseases (Tp), between new diseases and known miRNAs (Td), between known diseases and new miRNAs (Tm) and between new diseases and new miRNAs (Tn), respectively. Experimental results indicate that the proposed ReHoGCN(ES)-MDA method outperformed UReHoGCN(ES) and HeGCN(ES) across all four tasks, subtly highlighting that a steady degree distribution, significantly contributes to enhancing model performance. Furthermore, ReHoGCNES-MDA surpassed several machine learning algorithms and cutting-edge methods in MDA prediction. Additionally, three case studies were conducted for miRNAs and diseases, respectively, which also underline the satisfactory performance and validate the effectiveness of the proposed ReHoGCNES-MDA method. In sum, ReHoGCNES-MDA underscores the significance of a regular graph and can proficiently predict potential MDAs.

## MATERIALS AND METHODS

### Datasets

In our study, experimentally verified known MDAs were obtained from the Human-miRNA Disease Database (HMDD) v2.0 [72], released in 2014, encompassing 5430 associations between 495 miRNAs and 383 diseases. The subsequent release of HMDD v3.0 [73] in 2019 incorporated newly discovered MDAs, while the MDAs from HMDD v2.0 were designated as old. Known MDAs were regarded as positive samples, with the remaining were deemed indeterminate and categorized as unlabeled data, from which a number of pairs equivalent to the positive pairs were randomly chosen to form a negative dataset. Utilizing this approach, we devised five datasets for the first time, as detailed in Table 2 and Table S2: (i) the training set, comprising all old diseases, old miRNAs and their established association pairs; (ii) Tp test set, inclusive of all old diseases, old miRNAs and their newly identified association pairs; (iii) Td test set, containing all new diseases, old miRNAs and their interaction pairs; (iv) Tm test set, with all old diseases, new miRNAs and their association pairs; (v) Tn test set, encompassing all diseases, new miRNAs and their association pairs.

**Table 2:** Number of entries of the training set and four different test sets obtained from HMDD v2.0 and HMDD v3.0

Datasets	Training set	Test set			
		Tp	Td	Tm	Tn
Disease	383	383	474	383	474
miRNA	495	495	495	567	567
Associations	5430	3848	4200	2780	689

### Node feature representations

Integrated features based on the diseases semantic similarity, miRNAs functional similarity and Gaussian interaction profile (GIP) kernel similarities were adopted in this study.

#### Disease semantic similarity matrix

Utilizing the Medical Subject Headings descriptors [24], we calculated the Disease Semantic Similarity Matrix (DSSM), which is accessible at https://www.ncbi.nlm.nih.gov/. The Directed Acyclic Graph (DAG), illustrating the relationships among various diseases, has been extensively utilized in numerous studies [56, 74] to construct the DSSM. Two distinct DSSMs were defined based on two different rationales. DSSM_1_ is formulated under the premise that two diseases sharing a larger portion of their DAGs are more similar. DSSM_2_, on the other hand, posits that a disease occurring in more (or fewer) DAGs may be more common (or specific). To derive a more rational DSSM, element-wise averaging was conducted on the aforementioned DSSMs to amalgamate them into the final DSSM.

#### MiRNA functional similarity matrix

The construction of the miRNA Functional Similarity Matrix (MFSM) is predicated based on the assumption that miRNAs exhibiting similar functions are more prone to association with diseases showcasing similar phenotypes, and the converse holds true as well [74]. The gene–gene interaction data were procured from HumanNet [75], where each edge is ascribed a weight determined by a corresponding log-likelihood score. Initially, we applied Min-Max normalization to the scores. Subsequently, the functional similarity between any pair of genes is calculated as follows:


(1) \begin{equation*} S\left({g}_i,{g}_j\right)=\left\{\begin{array}{@{}c}0,\kern2.75em e\left({g}_i,{g}_j\right)\notin E\\{}1,\kern5.25em {g}_i={g}_j\\{}w\left({g}_i,{g}_j\right),\kern0.5em e\left({g}_i,{g}_j\right)\in E\end{array}\right. \end{equation*}

where $w\left({g}_i,{g}_j\right)$represents the normalized value transformed by Min-Max normalization, $E$ denotes the edges set in gene interaction network and $e\left({g}_i,{g}_j\right)$ denotes the edge.

For miRNAs *i* and *j*, ${G}_i$ represents a set of genes associated with miRNA *i*, and ${G}_j$ represents a set of genes associated with miRNA *j*, in which ${G}_i$ contains $\left|{G}_i\right|$ genes and ${G}_j$ contains $\left|{G}_j\right|$ genes. Then, the functional similarity of miRNAs ${m}_i$ and ${m}_j$ is calculated as follows:


(2) \begin{equation*} {\displaystyle \begin{array}{c} MFSM\left({m}_i,{m}_j\right)=\frac{\sum_{g\in{G}_i}\kern0.1em S\left(g,{G}_j\right)+\sum_{g\in{G}_j}\kern0.1em S\left(g,{G}_i\right)}{\left|{G}_i\right|+\left|{G}_j\right|}\ \end{array}} \end{equation*}

where $\left|G\right|$ is the cardinality of gene set $G$, and $S\left(g,G\right)=\underset{g_t\in G}{\mathit{\max}}\kern0.1em \left(S\left(g,{g}_t\right)\right)$. The MFSM is accessible at https://www.cuilab.cn/files/images/cuilab/misim.zip.

#### GIP kernel similarity

The GIP kernel similarity for miRNAs (diseases) was constructed based on the assumption that functionally (phenotypic) similar miRNAs (diseases) exhibit similar patterns with diseases (miRNAs) [76]. Taking the construction of GIP kernel similarity matrix (DGSM) as an example. First, for a given disease, we used vector $P\left({d}_i\right)$ to denote the interaction profile of this disease, which is a binary vector and corresponds to the $i$th row of miRNA-disease adjacency matrix. The values (‘1’ or ‘0’) in $P\left({d}_i\right)$ indicate whether disease has a known association with each miRNA or not. Then, we can obtain DGSM by calculating the similarity between each disease pair:


(3) \begin{equation*} {\displaystyle \begin{array}{c} DGSM\left({d}_i,{d}_j\right)=\exp \left(-{\gamma}_d{\parallel P\left({d}_i\right)-P\left({d}_j\right)\parallel}^2\right)\end{array}} \end{equation*}

(4) \begin{equation*} {\displaystyle \begin{array}{c} {\gamma}_d=\frac{\gamma_d^{\prime }}{\left(\frac{1}{n}\sum_{i=1}^n\kern0.1em {\parallel P\left({d}_i\right)\parallel}^2\right)}\end{array}} \end{equation*}

where $n$ represents the number of rows of the MDA matrix *A*, that is, the number of all miRNAs. ${\gamma}_d^{\prime }$ is a initial bandwidth parameter that can be determined by further cross-validation. According to previous research [76, 86], almost all researchers have simply set it to 1. The parameter ${\gamma}_d$ was then employed to regulate the kernel bandwidth. The GIP kernel similarity matrix for miRNAs (MGSM) can be calculated in a similar manner.

#### Integrating similarity for miRNAs and diseases

Given the prevalence of sparse values in the earlier obtained MFSM and DSSM, we amalgamated the GIP kernel similarity matrices MGSM and DGSM to address the zero-value entries, respectively. Consequently, we acquired the Integrated miRNA Similarity Matrix (IMSM) and Integrated Disease Similarity Matrix (IDSM). Utilizing IMSM as an illustration, the integrated equations [77] are:


(5) \begin{equation*} {\displaystyle \begin{array}{c} \mathrm{IMSM}\left({m}_i,{m}_j\right)=\left\{\begin{array}{@{}cc}\mathrm{MFSM}\left({m}_i,{m}_j\right)& \mathrm{MFSM}\left({m}_i,{m}_j\right)\ne 0\\{}\mathrm{MGSM}\left({m}_i,{m}_j\right)& \mathrm{otherwise}\ \end{array}\ \right.\end{array}} \end{equation*}

### ReHoGCNES model for predictions of MDAs

There are two crucial steps to build ReHoGCN model we proposed: (i) construction of regular graph by k-NN algorithm and (ii) predictions of MDAs using a novel GCN model via edge-based graph sampling.

#### Construct homogenous GCN with regular graph structure

Consider one layer GCN:


(6) \begin{equation*} {\displaystyle \begin{array}{c}{H}^{l+1}=\sigma \left({\overset{\sim }{D}}^{-\frac{1}{2}}\overset{\sim }{\mathrm{A}}{\overset{\sim }{D}}^{-\frac{1}{2}}{H}^l{W}^l\right)\end{array}} \end{equation*}

Here, $A$is denoted as an adjacency matrix of graph *G.* Generally, $\overset{\sim }{A}=A+{I}_N$ is used to replace with *A* by added self-connections, where${I}_N$ is the identity matrix. $\overset{\sim }{D}$ is the normalized degree matrix. $\sigma \left(\cdot \right)$ represents an activation function. The weight matrix ${W}^l$ is specific and trainable to each layer. ${\mathrm{H}}^l\in{\mathbb{R}}^{N\times D}$ is the matrix of activations in the *l*th layer.

Equation (6) can be divided into two steps: (i) first, a new feature matrix $Y$ was obtained by utilizing graph convolution,


(7) \begin{equation*} {\displaystyle \begin{array}{c} Y={\overset{\sim }{D}}^{-\frac{1}{2}}\overset{\sim }{\mathrm{A}}{\overset{\sim }{D}}^{-\frac{1}{2}}{H}^l{W}^l\kern0.75em \end{array}} \end{equation*}

(ii) Then add a fully connected layer on $Y$, ${H}^{l+1}=Y{W}^l.$

Symmetic normalized Laplacian matrix is ${L}^{sym}={D}^{-\frac{1}{2}}\mathrm{A}{D}^{-\frac{1}{2}}$. GCN can be regarded as a special form of Laplacian smoothing. Laplacian quadratic form can represent the smoothness of graph network structure. From the point of view of information aggregation, normalized Laplacian matrix is a weighted aggregation of the first-order neighbor information of a node, and the weight is inversely proportional to the degree of the node. A regular graph is a perfect fit with the Laplacian idea of smoothing, which is to make a point as similar as possible to the points around it, and the new feature of each node is the mean of the features of the nodes around it. The property of regular graph is able to let each node better use the information of the surrounding nodes.

The heterogeneity of biological data often poses challenges in obtaining regular graphs. To thoroughly encapsulate the structural information within the feature space, we construct adjacency matrix using the $k$-NN algorithm, focusing on the node feature and the topological relationships between miRNAs and diseases. This adjacency matrix represents the similarity or distance relationships between data points, with the KNN method retaining the K nearest neighbors for each data point. The specific steps are as follows: (i) compute the distance or similarity between data points to generate a distance matrix or similarity matrix. (ii) For each data point, select the K nearest points from its neighbors and set the corresponding row in the matrix to represent the neighbors of that data point. (iii) Set non-symmetric elements in the adjacency matrix to 0 and symmetric elements to the average of their distances or similarities. It is clear that $k$-NN algorithm is an efficient way to generate regular graphs because degree matrix of graph obtained by $k$-NN algorithm is $\mathit{\operatorname{diag}}\left(k,\dots, k\right)$. Given that the $k$-NN algorithm is applied across all data, we assign a label of 0 to nodes within the test set to ensure there’s no leakage of test data during the training phase. This graph construction approach facilitates the generation of a regular graph in a simple yet effective manner, enabling the optimal utilization of graph information.

In comparison, we also proposed a homogenous GCN with a non-regular graph structure (UReHoGCN) utilizing $k$-means algorithms, and a HeGCN. UReHoGCN replaces $k$-NN algorithm with $k$-means algorithms to generate different numbers of neighbors for each node. HeGCN consists of three interlinked sub-graphs whose adjacency matrices are disease-disease fully connected matrix, miRNA-miRNA fully connected matrix and miRNA-disease link matrix.

After parameter-tuning (Supplementary Figures S1 and S2), the best $k$ parameter is 5 for ReHoGCN model and the best *n* parameter is 300 for UReHoGCN model. Detailed degree statistics and degree distribution of the three proposed models are shown in Table 3 and Figure S3, respectively.

**Table 3:** Detailed degree statistics of the three proposed models

Models	Max node degree	Min node degree	Total node degree	Total number of nodes	Average node degree
ReHoGCN(ES)	5	5	54 330	10 860	5
UReHoGCN(ES)	198	5	280 605	10 860	$\approx 26$
HeGCN(ES)	54	0	10 860	865	$\approx 13$

#### ReHoGCN model with random ES

We transformed the MDA prediction task from link prediction to node prediction, which came with increased training complexity due to the growth in the number of nodes and edges within the graph. To address this issue, we employed a random ES strategy known for its scalability, efficiency and low training complexity, which also aids in averting the over-smoothing problem. A crucial consideration when defining an ES is to ensure that edges with non-negligible probability are sampled. Concurrently, normalization was carried out to reduce the variance in aggregated node information and mini-batch loss in full GCNs. The probability of an edge ($u,v$) being sampled in a subgraph was calculated as follows:


(8) \begin{equation*} {\displaystyle \begin{array}{c}p\left({e}_{u,v}\right)=\frac{\left(\frac{1}{\deg (u)}+\frac{1}{\deg (v)}\right)}{\sum_{\left({u}^{\prime },{v}^{\prime}\right)\in E}\kern0.1em \left(\frac{1}{\deg \left({u}^{\prime}\right)}+\frac{1}{\deg \left({v}^{\prime}\right)}\right)}\ \end{array}} \end{equation*}

where deg($u$) and deg($v$) mean the degree of node $u$ and $v$, respectively. $E$ is the set of edges and $\left({u}^{\prime },{v}^{\prime}\right)$ is an any edge in set $E.$$M$ edges randomly sampled (with replacement) from $E$ according to $p.$ From Equation (8), each edge has non-negligible probability to be sampled. However, this sampler that preserves connectivity characteristic of graph $G$ will almost inevitably introduces bias into minibatch estimation. Here, we present normalization techniques to eliminate biases.

Normalization coefficients ${\alpha}_{u,v}$ and ${\lambda}_v$ are defined as follows:


$$ {\alpha}_{u,v}=\frac{C_{u,v}}{C_v}=\frac{C_{v,u}}{C_v}\ \mathrm{and}\ {\lambda}_v=\frac{C_v}{N} $$

where ${C}_v$ denotes the frequency of node ${v}^{\prime}\mathrm{s}$ appearance within the $N$ subgraphs and ${C}_{u,v}$ denotes the frequency of edge ($u,v$)’s appearance within the $N$ subgraphs.

For a regular graph, ${p}_e^{(l)}=p.$For a previously sampled ${u}^{(l)}$ to establish a connection to layer $l+1$, at least one of its edges has to be selected by the layer $l+1$ sampler. It is obvious that the probability of a node in input layer survive $N$ number of independent sampling process is ${\left(1-{\left(1-p\right)}^d\right)}^{L-1}$. Such layer sampler may yield an overly sparse minibatch for $L>1.$Moreover, the connectivity within a minibatch remains unaffected with the depth of GCN, implying that if an edge is present in layer $l$, it persists through all layers. During propagation within the GCN layer, precise node embedding can be derived within subgraphs, and sampled nodes can mutually reinforce each other without requiring external information from outside the batch. This methodology naturally alleviates the neighbor explosion dilemma, typically faced by GCN algorithms. Additionally, the preprocessing overhead is minimal as all subgraphs can be utilized as minibatches throughout the training phase.

### Overall approach and experimental execution

**Figure 2:**
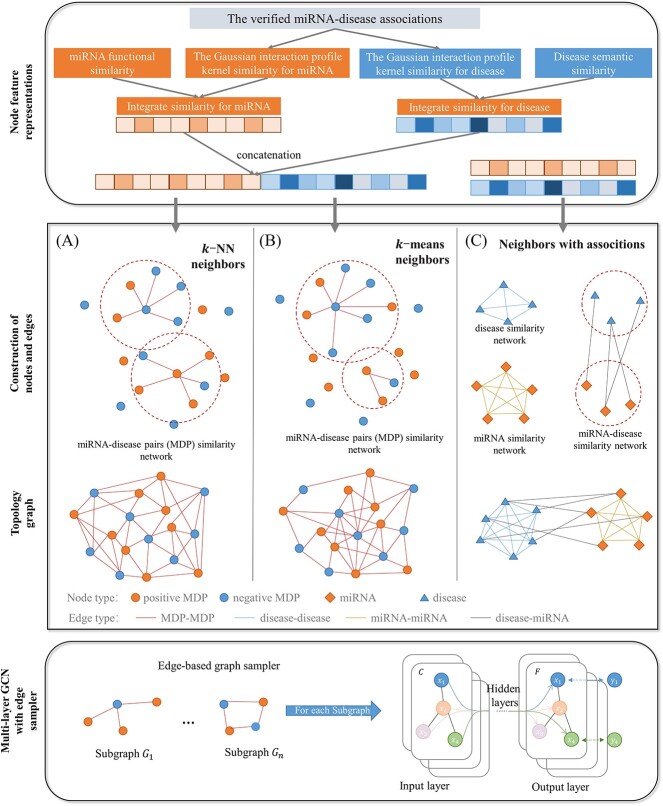
The workflow of the proposed method, where the MDP represents the miRNA-disease pair, GCN represents GCN. Three graph architectures of GCN model were proposed in our work. (a) Homogenous GCN with regular graph structure through the *k*-NN algorithm (ReHoGCN model); (b) homogenous GCN with non-regular graph structure (UReHoGCN model) through *k*-means algorithms; (c) traditional HeGCN (HeGCN model). IDSM and IMSM are concatenated as node features for ReHoGCN and UReHoGCN model, while IDSM and IMSM are features of disease-disease similarity network and miRNA-miRNA similarity network in parallel for HeGCN model. Then, an ES was used for GCN training process.

Figure 2 depicted the workflow of the proposed method. First, IDSM and IMSM are calculated as integrated node features of miRNA and disease. Next, three graph architectures of GCN model were proposed in our work. (a) Homogenous GCN with regular graph structure through the *k*-NN algorithm (ReHoGCN model); (b) homogenous GCN with non-regular graph structure (UReHoGCN model) through *k*-means algorithms; (c) traditional HeGCN (HeGCN model). IDSM and IMSM are concatenated as node features for ReHoGCN and UReHoGCN model, while IDSM and IMSM are features of disease-disease similarity network and miRNA-miRNA similarity network in parallel for HeGCN model. Then, an ES was used for GCN training process. Comprehensive data regarding the hyperparameters and architectures of the ReHoGCNES model (ReHoGCN model with random ES) are provided in Supplementary Table S3. Table 4 listed experimental environment like hardware environment, software environment, program languages and libraries used in our work. And, the data and source codes are available from https://github.com/yufangz-sjtu/ReHoGCNES-MDA to use this architecture and reproduce the results.

**Table 4:** Experimental environment

Hardware environment	CPU Intel(R) Xeon(R) Gold 6230 CPU @ 2.10GHz	
Software environment	Operating system	CentOS Linux release 7.7.1908 (Core)
	Program language	Python 3.9.0
	Libraries	Numpy 1.19.2 Scikit-learn 1.0.1 Pandas 1.3.4 Torchvision 0.13.1 Scipy 1.6.2 Cython 0.29.23

All experiments were conducted 10 times to ascertain average scores of the prediction outcomes. The evaluation metrics for the model encompass accuracy, precision, recall, F1-score and the area under the Receiver Operating Characteristic curve (AUC).

## RESULTS

### Prediction performance of three graph architecture

Model evaluation metrics of ReHoGCN and comparisons with other proposed models UReHoGCN and HeGCN in this work is shown in Table 5. Table 5 illustrated that ReHoGCN with regular graph structure achieved better AUC than unregular graph structure UReHoGCN and HeGCN on both four MDP prediction tasks. Besides, UReHoGCN achieved second best AUC on MDA predictions. This shows that taking associations among MDPs into consideration is an easy and effective way to the fusion of heterogeneity. The predicament of heterogeneous graph network like HeGCN is paying attention to the topology structure information of the graph, such as different types of points and edges and the attributes of each node at the same time. Homogenous GCN with regular graphs have achieved certain advantages in MDA prediction problems and can also provide useful insights for other tasks. *P*-values are obtained by conducting a paired *t*-test between the AUC of the three proposed model on four tasks, which are presented in Table 6. The results are very small indicate the statistical significance of improvements.

**Table 5:** Prediction performance of our proposed methods ReHoGCN compared with UReHoGCN and HeGCN without ES on four tasks

Task	Model	Accuracy	Precision	Recall	F1-Score	AUC
Tp	ReHoGCN	0.9954	1.0000	0.9906	0.9953	0.9996
	UReHoGCN	0.9648	0.9695	0.9603	0.9643	0.9920
	HeGCN	0.9277	0.9240	0.9273	0.9312	0.9650
Td	ReHoGCN	0.9788	0.9912	0.9678	0.9798	0.9985
	UReHoGCN	0.9588	0.9536	0.9559	0.9500	0.9839
	HeGCN	0.9275	0.9430	0.9297	0.9273	0.9489
Tm	ReHoGCN	0.9865	0.9924	0.9823	0.9854	0.9985
	UReHoGCN	0.9451	0.9404	0.9394	0.9448	0.9825
	HeGCN	0.9322	0.9342	0.9379	0.9287	0.9540
Tn	ReHoGCN	0.9567	0.9742	0.9422	0.9570	0.9940
	UReHoGCN	0.9427	0.9480	0.9489	0.9446	0.9734
	HeGCN	0.9247	0.9286	0.9293	0.924	0.9372

**Table 6:** Two-tailed *P*-values of paired *t*-test for AUC on four datasets

Paied model	Tp	Td	Tm	Tn
ReHoGCN-UReHoGCN	2.660162e-04	4.98735e-06	2.11492e-06	6.80115e-08
ReHoGCN-HeGCN	1.203205e-08	3.29430e-09	3.11945e-09	1.28779e-09
UReHoGCN-HeGCN	4.464112e-06	5.61764e-07	2.71103e-07	1.96400e-60

According to different tasks, all three GCN models have achieved the best model performance and obtain the highest AUC, accuracy, precision, recall and F1-Score on the Tp task, especially ReHoGCN. The test set Tp contains the associations of old miRNA, old diseases, that is, the miRNA or diseases in test set Tp are all included in the training phase while the other test sets contain new miRNA or new diseases or both new. ReHoGCN models also achieve good results on the completely new dataset Tn, which shows that it has good predictive ability for new data. The robustness and scalability of the model is validated. Compared with task Tp, the accuracy of the model on task Tn is reduced. This is because the construction of our negative sample is randomly selected from the unknown MDAs. There are many potential MDAs between them. Due to the reduction of samples, the false negative problem in the sample is very prominent. In addition, the distribution of test sets is very uneven, and the distribution compared with the training set is deviated, which will also affect the accuracy of the model.

### Prediction performance of ReHoGCN compared with UReHoGCN and HeGCN with/without random ES

**Figure 3:**
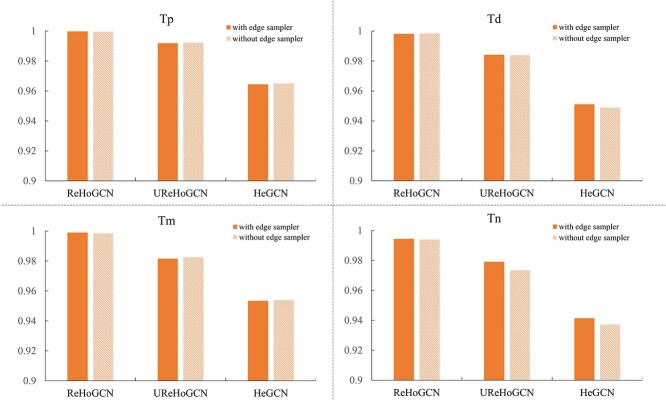
AUC of three graph construction methods with/without ES on four tasks.

**Figure 4:**
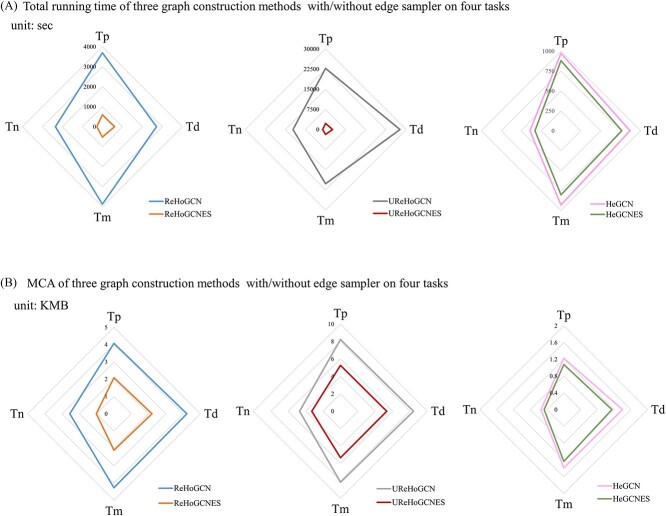
Analysis of the influence of random ES from two aspects. (a) Total training time of three graph construction methods with/without ES on four tasks; (b) MAC of three graph construction methods with/without ES on four tasks.

Although ReHoGCN achieved better prediction performance than HeGCN, it increases the node numbers and degrees leading to enormous computation complexity and memory access cost (MAC). ES is helpful to solve these problems (Supplementary Table S4). Figures 3 and 4 show that random ES can significantly reduce the training time (numerical value and *P*-value, Supplementary Tables S5 and S8) and MAC (numerical value and *P*-value, Supplementary Tables S6 and S8) with no loss of prediction accuracy (numerical value and *P*-value, Supplementary Tables S4 and S7) on model ReHoGCN and UReHoGCN. ES defines ‘influence’ from the graph connectivity perspective and considers joint information from node connections as well as node attributes. Only nodes having high influence are sampled by this sampler instead of information aggregation on full graph, which will avoid ‘Neighbor Explosion’ phenomena or over-smoothing problem. This ES has been proven to be unbias and has minimal variance contributed to its satisfying model performance. However, on the HeGCN model, we did not observe the same results as above. One possible reason is that with small number of nodes and links of the graph, the operation time and MAC proportion of sampling probability calculation and subgraph generation are not negligible relative to the entire full-graph calculation (detailed analysis, Supplementary Table S9). Therefore, the advantages of subgraph sampling are not shown. For subsequent comparison with other methods, we select the ReHoGCNES model as the best prediction performance. In conclusion, ReHoGCNES has three advantages: (i) high accuracy, (ii) high connectivity and efficiency and (iii) low training complexity.

### Performance of ReHoGCNES model compared with the state-of-the-art network-based methods

In order to further prove the superiority of the proposed ReHoGCNES method, we compare it with five state-of-the-art network-based methods published after 2022, including LAGCN [78], DEJKMDR [79], NSAMDA [80], HGANMDA [81] and HLGNN-MDA [82] under the same experimental conditions. The aforementioned methods applied varieties of graph construction strategies: (i) LAGCN firstly integrates three associations into a heterogeneous network and applies GCN with attention mechanisms to learn the embedding of miRNA and disease. (ii) The DEJKMDR is a HeGCN model which randomly deletes edges to increase the diversity of data and reduces overfitting. (iii) NSAMDA identified the MDAs based on neighbor selection graph attention networks. (iv) HGANMDA constructed a miRNA-disease-lncRNA heterogeneous graph and node-layer attention was applied to learn the importance of neighbor nodes based on different meta-paths. (v) HLGNN-MDA proposes heuristic learning network enabling it to learn information among homogenous and heterogeneous nodes. We verified the superiority of the ReHoGCNES method on the four datasets and Table 7 shows that ReHoGCNES is better than the five state-of-the-art methods on all four datasets especially on completely new dataset Tn, which shows that ReHoGCNES has good predictive ability for new data. Table 8 shows *P*-values of paired *t*-test for AUC between ReHoGCNES model and on four datasets. All *P*-values are far <0.01 meaning that the difference probability among models due to sampling error is <0.01 and model improvement by ReHoGCNES is statistical significant. ReHoGCNES combined beneficial similarity features to build a homogenous network, thereby maximizing the utility of available information through the aggregation of neighborhood data. And, ReHoGCNES utilized regular graphs for the GCN model which offers advantages in terms of accuracy and scalability.

**Table 7:** Results of ReHoGCNES performance compared with five state-of-the-art models since 2022 on four tasks

Task	Model	Accuracy	Precision	Recall	F1-Score	AUC
Tp	ReHoGCNES	0.9957	0.9979	0.9936	0.9957	0.9998
	LAGCN	0.8436	0.8315	0.8273	0.8302	0.9202
	DEJKMDR	0.8512	0.8123	0.9124	0.8593	0.9576
	NSAMDA	0.8976	0.9037	0.8864	0.8968	0.9169
	HGANMDA	0.8628	0.8589	0.8687	0.8636	0.9045
	HLGNN-MDA	0.9402	0.9547	0.9245	0.9433	0.9559
Td	ReHoGCNES	0.9799	0.9909	0.9688	0.9797	0.9982
	LAGCN	0.8034	0.8054	0.8388	0.8149	0.9062
	DEJKMDR	0.8398	0.8235	0.846	0.8332	0.9276
	NSAMDA	0.8565	0.8564	0.8348	0.8576	0.8733
	HGANMDA	0.8379	0.8203	0.8467	0.8432	0.8674
	HLGNN-MDA	0.9045	0.9217	0.8946	0.9235	0.9303
Tm	ReHoGCNES	0.9870	0.9931	0.9808	0.9869	0.9990
	LAGCN	0.8638	0.8762	0.8497	0.8643	0.9067
	DEJKMDR	0.9043	0.8879	0.9166	0.9099	0.9556
	NSAMDA	0.8867	0.8453	0.8991	0.8805	0.9265
	HGANMDA	0.8554	0.8279	0.8356	0.8581	0.8997
	HLGNN-MDA	0.9174	0.9293	0.9163	0.9232	0.9486
Tn	ReHoGCNES	0.9581	0.9751	0.9402	0.9573	0.9944
	LAGCN	0.6243	0.6055	0.6187	0.6184	0.6634
	DEJKMDR	0.7468	0.7401	0.7386	0.7422	0.7693
	NSAMDA	0.7258	0.7047	0.7223	0.7279	0.7374
	HGANMDA	0.6472	0.6584	0.6395	0.6442	0.6987
	HLGNN-MDA	0.7789	0.7654	0.7801	0.7793	0.8042

**Table 8:** Two-tailed *P*-values of paired *t*-test between the AUC of the ReHoGCNES and other state-of-the-art models on four tasks

Paied model	Tp	Td	Tm	Tn
LAGCN	1.12555e-11	6.13011e-12	2.58717e-10	6.03358e-14
DEJKMDR	2.04118e-09	2.41621e-06	2.72648e-09	9.18840e-10
NSAMDA	3.29359e-10	2.60846e-11	1.62319e-10	6.15496e-10
HGANMDA	2.23766e-10	8.69261e-11	1.13554e-10	6.32573e-10
HLGNN-MDA	2.01516e-09	3.41725e-10	1.16653e-09	2.54908e-09

### Performance comparison between ReHoGCNES and classic machine learning and deep learning models

**Figure 5:**
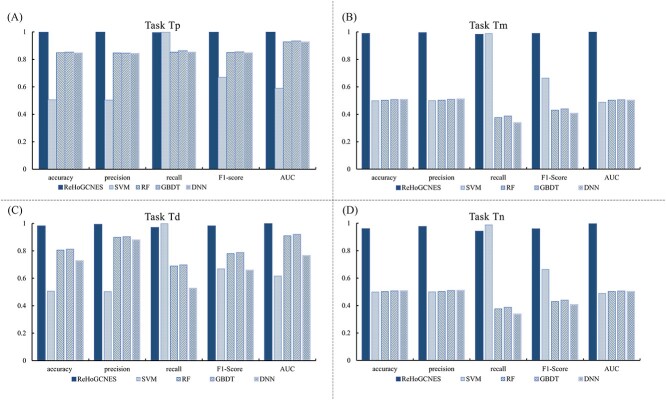
Results of ReHoGCNES performance compared with classic machine learning and deep learning models on four test sets ((a) Tp, (b) Tm, (c) Td, (d) Tn) obtained from HMDD.

We conducted a comparative analysis of the ReHoGCNES model against RF, Support vector machine (SVM), Deep Neural Network (DNN) and Gradient Boosting Decision Tree (GBDT) to show its superiority in MDA prediction. The results, presented in Figure 5 and Table S10 showcase that our proposed model is superior to other traditional ML and DL models. The AUCs of our method are higher than SVM, GBDT, RF and DNN by 70.64, 7.12, 7.79 and 7.89%, respectively. Table 9 shows two-tailed *P*-values of paired *t*-test between the AUC of the ReHoGCNES and machine learning and deep learning models on four tasks indicating the statistical significance of improvements. The excellent performance of ReHoGCNES is attributed to its effective and powerful graph processing ability via adaptive neighbor feature aggregation. Compared with DNN models, ReHoGCNES proves more suitable for handing graph-structured data like MDA predictions. Although tree-based methods like GBDT and RF models outperformed DNN, it is worth to note that there is a chance of improving the predictive performance of the DNN model by increasing the number of hidden layers since we’ve fine-tuned the parameters on the default number of network layers. Both ML and DL models failed to predict new MDPs. Both GCN-based method requires newly discovered both miRNAs and diseases in the graph composition process, which is impossible to achieve in the real world. Therefore, although ReHoGCNES achieved better performance in the Tn dataset, it still has limitations for associations between new miRNAs and new diseases.

**Table 9:** Two-tailed *P*-values of paired *t*-test between the AUC of the ReHoGCNES and machine learning and deep learning models on four tasks

Paied model	Tp	Td	Tm	Tn
SVM	2.14803e-12	2.08277e-12	2.08184e-13	1.22925e-14
RF	1.08819e-08	3.69237e-10	1.35486e-08	1.00977e-13
GBDT	1.54394e-08	1.77081e-06	6.03405e-09	2.56388e-13
DNN	1.02950e-08	1.02282e-12	2.40499e-09	2.10407e-13

### Case studies

We employed the devised method ReHoGCNES to predict new MDAs for three prevalent human diseases (breast neoplasms, prostate neoplasms, pancreatic neoplasms), leveraging the known associations from HMDD. Our method was executed to ascertain the prediction scores of candidate miRNAs in relation to these neoplasms. The scores of the candidate associations were ranked, and the top 30 candidate associations with these diseases were selected. Subsequently, the prediction results were validated by two databases: dbDEMC V3.0 [83] and PhenomiR [84]. The results from the three case studies are detailed in Tables 10–12. Consequently, 28 of the top 30 miRNAs were confirmed to be associated with breast neoplasms, 27 of the top 30 miRNAs with prostate neoplasms, and 28 of the top 30 miRNAs with pancreatic neoplasms. These findings confirm that our method is capable of effectively predicting potential MDAs. Table 13 shows comparison between ReHoGCNES and five state-of-the-art models about predicted miRNAs associated with three kinds of neoplasms. $N$of top $30$ new MDAs are confirmed by different methods. And we calculate overlap ratio between MDAs found by proposed ReHoGCNES method and other methods.

**Table 10:** The top 30 predicted miRNAs associated with breast neoplasms

miRNA(1–15)	Evidence	miRNA(16–30)	Evidence
hsa-mir-542	dbDEMC;PhenomiR	hsa-mir-615	dbDEMC;PhenomiR
hsa-mir-211	dbDEMC;PhenomiR	hsa-mir-142	dbDEMC;PhenomiR
hsa-mir-449a	dbDEMC;PhenomiR	hsa-mir-15b	dbDEMC;PhenomiR
hsa-mir-98	dbDEMC;PhenomiR	hsa-mir-454	dbDEMC;
hsa-mir-99b	dbDEMC;PhenomiR	hsa-mir-138-2	dbDEMC;PhenomiR
hsa-mir-92b	dbDEMC;PhenomiR	hsa-mir-138-1	dbDEMC;PhenomiR
hsa-mir-192-2	Unconfirmed	hsa-mir-330	dbDEMC;PhenomiR
hsa-mir-494	dbDEMC;PhenomiR	hsa-mir-378a	dbDEMC;PhenomiR
hsa-mir-28	dbDEMC	hsa-mir-28	dbDEMC;PhenomiR
hsa-mir-106a	dbDEMC;PhenomiR	hsa-mir-186	dbDEMC;PhenomiR
hsa-mir-95	dbDEMC;PhenomiR	hsa-mir-219-2	dbDEMC;PhenomiR
hsa-mir-211	dbDEMC;PhenomiR	hsa-mir-99a	dbDEMC;PhenomiR
hsa-mir-19b-2	dbDEMC	hsa-mir-683	Unconfirmed
hsa-mir-29b	dbDEMC;PhenomiR	hsa-mir-128	dbDEMC;PhenomiR
hsa-mir-185	dbDEMC;PhenomiR	hsa-mir-153-1	dbDEMC;PhenomiR

**Table 11:** The top 30 predicted miRNAs associated with prostate neoplasms

miRNA(1–15)	Evidence	miRNA(16–30)	Evidence
hsa-mir-9-3	dbDEMC;PhenomiR	hsa-let-7a-3	dbDEMC;PhenomiR
hsa-mir-191	dbDEMC;PhenomiR	hsa-let-7f-1	dbDEMC;PhenomiR
hsa-let-7f-2	dbDEMC;PhenomiR	hsa-let-7a-2	dbDEMC;PhenomiR
hsa-mir-142	dbDEMC;PhenomiR	hsa-mir-9-1	dbDEMC;PhenomiR
hsa-let-7b	dbDEMC;PhenomiR	hsa-mir-181b-1	dbDEMC;PhenomiR
hsa-mir-29b	dbDEMC;PhenomiR	hsa-mir-29b	Unconfirmed
hsa-mir-10	dbDEMC;PhenomiR	hsa-mir-218-2	dbDEMC;PhenomiR
hsa-mir-7-3	dbDEMC;PhenomiR	hsa-mir-7-1	dbDEMC;PhenomiR
hsa-mir-215	dbDEMC;PhenomiR	hsa-mir-95	Unconfirmed
hsa-mir-139	dbDEMC;PhenomiR	hsa-mir-7	dbDEMC;PhenomiR
hsa-mir-181b-2	dbDEMC;PhenomiR	hsa-mir-7-2	dbDEMC;PhenomiR
hsa-mir-103a-2	dbDEMC	hsa-mir-221	dbDEMC
hsa-mir-201	dbDEMC;PhenomiR	hsa-mir-9-2	dbDEMC;PhenomiR
hsa-mir-218-1	dbDEMC;PhenomiR	hsa-let-7 g	dbDEMC;PhenomiR
hsa-mir-99a	Unconfirmed	hsa-mir-9-3	dbDEMC;PhenomiR

**Table 12:** The top 30 predicted miRNAs associated with pancreatic neoplasms

miRNA(1–15)	Evidence	miRNA(16–30)	Evidence
hsa-mir-296	dbDEMC;PhenomiR	hsa-mir-101-2	dbDEMC;PhenomiR
hsa-mir-100	dbDEMC;PhenomiR	hsa-mir-93	Unconfirmed
hsa-mir-143	dbDEMC;PhenomiR	hsa-mir-429	dbDEMC;PhenomiR
hsa-mir-132	dbDEMC;PhenomiR	hsa-let-7a-1	Unconfirmed
hsa-mir-101	dbDEMC;PhenomiR	hsa-let-7a-2	dbDEMC;PhenomiR
hsa-mir-103a-1	dbDEMC;PhenomiR	hsa-mir-138-2	dbDEMC;PhenomiR
hsa-mir-425	dbDEMC;PhenomiR	hsa-mir-10b	dbDEMC;PhenomiR
hsa-mir-212	dbDEMC;PhenomiR	hsa-mir-210	dbDEMC;PhenomiR
hsa-mir-218-2	dbDEMC;PhenomiR	hsa-mir-107	dbDEMC;
hsa-mir-27a	dbDEMC;PhenomiR	hsa-mir-10a	dbDEMC;PhenomiR
hsa-mir-138-1	Unconfirmed	hsa-mir-200c	dbDEMC;PhenomiR
hsa-mir-216a	dbDEMC;PhenomiR	hsa-mir-218-1	dbDEMC;PhenomiR
hsa-mir-224	dbDEMC;PhenomiR	hsa-mir-10a	dbDEMC;PhenomiR
hsa-mir-200a	dbDEMC;PhenomiR	hsa-mir-95	dbDEMC;PhenomiR
hsa-let-7a-3	dbDEMC;PhenomiR	hsa-mir-196	dbDEMC;PhenomiR

**Table 13:** Comparison between ReHoGCNES and five state-of-the-art models about predicted miRNAs associated with three kinds of neoplasms. The $n$ column represents $n$ of top $30$ new MDAs are confirmed. The overlap ratio represents the repetition rate between associations found by other methods and proposed ReHoGCNES method

Disease	Item	ReHoGCNES	LAGCN	DEJKMDR	NSAMDA	HGANMDA	HLGNN-MDA
Breast neoplasms	*n*	28	25	28	26	26	28
	Overlap ratio	100%	56.67%	63.33%	66.67%	73.33%	83.33%
Prostate neoplasms	*n*	27	24	27	26	24	27
	Overlap ratio	100%	56.67%	63.33%	73.33%	70%	80%
Pancreatic neoplasms	*n*	28	26	25	25	25	27
	Overlap ratio	100%	60%	73.33%	76.67%	80%	86.67%

## DISCUSSION

In this paper, we first proposed a novel GCN-based graph-building strategy method (ReHoGCNES) based on regular graph with random ES to predict MDAs. The experimental results on four datasets demonstrate that the proposed ReHoGCNES-MDA method has achieved excellent results, which implicitly reveal steadily that degree distribution like uniform distribution of a graph does play an important role in enhancement of prediction performance. The robust performance of the proposed ReHoGCNES method can be attributed to several crucial factors. First, we integrated beneficial similarity features to build a homogenous network, thereby maximizing the utility of available information through the aggregation of neighborhood data. Second, we employed an inventive graph construction technique by utilizing regular graphs for the GCN mode. A range of experiments illustrated that regular graphs offer advantages in terms of graph connectivity, edge connectivity and subgraphs, which has advantages in accuracy, scalability and training complexity. The connectivity of the graph is an important indicator to measure its network resilience, which can allow the features of the central node to propagate to the spatial neighbors more effectively to obtain better model performance. In addition, the GCN’s Laplacian matrix eigenvalues have a very large relationship with the connectivity of the graph, and the second eigenvalue of the matrix is separately named the algebraic connectivity of the graph (Supplementary Proofs A). As far as we know, this is the first study to explore and compare the graph structure construction of different biological heterogeneous data, and the three different graph structures have achieved different results, which prove that not only the model parameters should be optimized in the model design stage, but also how to build graph is also crucial. Last but not least, the random ES facilitates efficient model operation while maintaining prediction performance. The continuous expansion of MDA datasets came with increased training complexity due to the growth in the number of nodes and edges within the graph. A random ES strategy employed in our study has potential in increasing efficiency, averting over-smoothing and reducing training complexity, which is helpful for GCN training process especially in large datasets. Additionally, this method holds promise for predicting related miRNAs of diseases even in the absence of known associations. In sum, ReHoGCNES-MDA highlights the significance of regular graphs and effectively predicts potential MDAs.

Nevertheless, our method has certain limitations. The datasets used for network construction may encompass noise and outliers. Additionally, the performance of the ReHoGCNES model warrants further validation with larger number of samples. Hence, our further research endeavors will be directed toward model validation utilizing more refined data.

**Key Points**

- The detection of miRNA-disease linkages via computational techniques utilizing biological information has emerged as a cost-effective and highly efficient approach.
- Three GCNs of different structures were tested on four experimental tasks on the MDA prediction problem for the first time. ReHoGCN model (homogenous GCN with regular graph structure through the *k*-NN algorithm) achieved best performances.
- The random ES implemented on ReHoGCN model can significantly reduce the training time and MAC with no loss of prediction accuracy.
- ReHoGCNES model that integrated beneficial similarity features and aggregated neighborhood data can offer advantages in terms of accuracy and scalability.

## Supplementary Material

final_SI_bbae103

## Data Availability

The data and source codes are available from https://github.com/yufangz-sjtu/ReHoGCNES-MDA.

## Author Biographies

**Yufang Zhang** is a PhD candidate at the School of Mathematical Sciences and SJTU-Yale Joint Center for Biostatistics and Data Science, Shanghai Jiao Tong University. She works on drug discovery and diseases-biomarker associations through machine learning models and statistic methods.

**Yanyi Chu** is a postdoctoral scholar at the Department of Pathology, Stanford University School of Medicine. Her research interests lie in miRNA vaccine design and drug-target interactions.

**Shenggeng Lin** is a master at the School of Life Sciences and Biotechnology, Shanghai Jiao Tong University. His major research interests include the predictions of drug–drug interactions and molecular generation.

**Yi Xiong** is an associate professor at the School of Life Sciences and Biotechnology, Shanghai Jiao Tong University. His main research interests focus on machine learning algorithms, and their applications in protein sequence–structure–function relationships and biomedicine.

**Dong-Qing Wei**, professor of bioinformatics, made many groundbreaking contributions to the development of bioinformatics techniques and their interdisciplinary applications to systems of ever-increasing complexity and published >520 papers, with 9299 citations and an H factor of 55.
