# Supplementary material for: ReHoGCNES-MDA: prediction of miRNA-disease associations using homogenous graph convolutional networks based on regular graph with random edge sampler
Source: Brief Bioinform. 2024 Mar 19;25(2):bbae103. doi: 10.1093/bib/bbae103 (PMC10959163; doi:10.1093/bib/bbae103)
Supplement: final_SI_bbae103 [file final_si_bbae103.docx]

**ReHoGCNES-MDA:** **prediction of miRNA-disease associations using homogenous graph convolutional networks based on regular graph with random edge sampler**

Yufang Zhang^1,2,3^, Yanyi Chu^4^, Shenggeng Lin^5^, Xiong Yi^5,6*^, Dong-Qing Wei^2,3,5^*

^1^School of Mathematical Sciences and SJTU-Yale Joint Center for Biostatistics and Data Science, Shanghai Jiao Tong University, Shanghai 200240, China

^2^Peng Cheng Laboratory, Shenzhen, Guangdong 518055, China

^3^Zhongjing Research and Industrialization Institute of Chinese Medicine, Zhongguancun Scientific Park, Meixi, Nanyang, Henan, 473006, China

^4^Department of Pathology, Stanford University School of Medicine, Stanford, CA, 94305, USA

^5^State Key Laboratory of Microbial Metabolism, School of Life Sciences and Biotechnology, and Joint Laboratory of International Cooperation in Metabolic and Developmental Sciences, Ministry of Education, Shanghai Jiao Tong University, Shanghai 200240, China

^6^Shanghai Artificial Intelligence Laboratory, Shanghai, 200232, China

* Corresponding author: [xiongyi@sjtu.edu.cn](mailto:xiongyi@sjtu.edu.cn); [dqwei@sjtu.edu.cn](mailto:dqwei@sjtu.edu.cn)

**Table S1** Summary of computational methods for miRNA-disease association prediction.

| **Algorithms** | **Similarity network** | **Training Datasets** | **Methods** | **Advantages** | **Disadvantages** | **Webserver/Website** |  |  |  |  |  |  |
| --- | --- | --- | --- | --- | --- | --- | --- | --- | --- | --- | --- | --- |
| **Similarity-based methods** | | | | | | |  |  |  |  |  |  |
| DNRLMF-MDA[1] | MFSM, DSSM, GIP kernel similarity for diseases/miRNA | HMDD2.0 | Logistic matrix factorization | Further improve prediction performance via dynamic  neighborhood regularized. | Model performance needs to be improved | NA |  |  |  |  |  |  |
| ELLPMDA[2] | MFSM, DSSM, GIP kernel similarity for diseases/miRNA | HMDD v2.0 | Ensemble Learning  Link Prediction  Similarity network | Suitable for diseases without any known related miRNAs. | Need to optimize parameter. | NA |  |  |  |  |  |  |
| HGIMDA[3] | MFSM, DSSM, GIP kernel similarity for diseases/miRNA | HMDD v2.0 | Iterative Multiplication of Similarity Matrix | This method could be effectively applied to new diseases and new miRNAs without any known associations. | Model prediction relies on known association relationships, and the optimal parameters have not been determined. | NA |  |  |  |  |  |  |
| GSTRW[4] | MiRNA family information,  MiRNA functional similarity,  Disease phenotypic similarity | gold benchmark data | Global similarity calculation based on the Laplacian score of graphs  two-tier network random walk | Take full advantage of  data | Many parameters need to be set. | NA |  |  |  |  |  |  |
| AMVML[5] | HMDD v2.0  MiRNA sequence similarity (pairwise Alignment)  MiRNA functional similarity  MiRNA semantic similarity(gene)  Disease semantic similarity 1  Gaussian interaction profile kernel similarity for diseases/miRNA | dbDEMC  miR2Disease | Similarity information self-conducted weight learning | Easy to implement and reliable | The value of the parameter has an impact on the prediction performance. | https://github.com/alcs417/AMVML |  |  |  |  |  |  |
| **Machine learning-based method** | | | | | | |  |  |  |  |  |  |
| RKNNMDA[6] | MFSM, DSSM and GIP kernel similarity for diseases/miRNA | HMDD v2.0 | Ranking-based KNN  Hamming Loss SVM Ranking | This method can be applied to diseases without any known related miRNAs. | This method is more biased towards well-known related diseases. | NA |  |  |  |  |  |  |
| RBMMMDA[7] | miRNA-target interactions, circulation, epigenetics and genetics | HMDD v2.0 | Restricted Boltzmann Machine | This model can predict new miRNA-disease associations as well as the types of associations. | This method cannot be used to predict isolated nodes and does not use any other useful biological information. | NA |  |  |  |  |  |  |
| EGBMMDA[8] | MFSM, DSSM1, DSSM2 | HMDD v2.0 | XGBoost | High accuracy, reliable and stable | This method cannot to predict new and isolated diseases/miRNA | http://www.escience.cn/system/file?fileId=91170^#^ |  |  |  |  |  |  |
| RFMDA[9] | MFSM, DSSM1, DSSM2, and  GIP kernel similarity for diseases/miRNA | HMDD v.2.0 | Select positive samples and negative samples  Select feature  Random Forest Regression | Simple to implement | This method cannot to predict new and isolated diseases/miRNA | NA |  |  |  |  |  |  |
| LMTRDA[10] | MFSM,miRNA sequences similarity(word2vec), DSSM1, DSSM2, and  GIP kernel similarity for diseases/miRNA | HMDD v3.0 | Multi-source feature fusion  Logistic model trees (LMT) classifier | The sequence information of miRNAs may be beneficial to predict potential miRNA-disease association for new miRNAs | Model generalization ability needs to be improved | NA |  |  |  |  |  |  |
| ABMDA[11] | MFSM, DSSM1, DSSM2, and  GIP kernel similarity for diseases/miRNA | HMDD v.2.0 | Selecting Negative Samples by Clustering  Adaboosting | Stable and reliable | Negative sample selection needs to be improved | NA |  |  |  |  |  |  |
| SDMMDA[12] | MFSM, DSSM1, DSSM2, and  GIP kernel similarity for diseases/miRNA | HMDD v2.0 | super-miRNAs/disease  multi-label K nearest neighbours algorithm | Stable and reliable | The aggregation method used has a certain degree of variability, which may introduce noise to the experimental data and affect the prediction results. | NA |  |  |  |  |  |  |
| **Deep learning-based methods** | | | | | | |  |  |  |  |  |  |
| DeepMDA[13] | MFSM, DSSM,  GIP kernel similarity for diseases/miRNA,  miRNA-target association (miRTarBase),  lncRNA-disease association, (LncRNADisease)  gene-disease association (DisGeNET) | HMDD v2.0 | Stacked autoencoder  Deep neural network | Integrate multiple data sets related to miRNA or diseases, and extract high-level features. | The data used are incomplete. | https://laiyifu.shinyapps.io/DeepMDA/ |  |  |  |  |  |  |
| AEMDA[14] | DSSM,  MFSM and GIP  kernel similarity for diseases/miRNA | HMDD v2.0 | Deep Auto Encoder | Simple network structure | Need integrating more data | https://github.com/CunmeiJi/AEMDA |  |  |  |  |  |  |
| BRWHNHA[15] | DSSM,  MFSM and GIP  kernel similarity for diseases/miRNA for diseases/miRNA | HMDD v2.0 | hybrid recommendation algorithm algorithm, which combined the heat spreading (HeatS) algorithm and probabilistic spreading (ProbS) algorithm  Restart random walk | Simple to implement. | This method has many parameters. | https://github.com/myl446/BRWHNHA |  |  |  |  |  |  |
| DBN-MF[16] | HMDD/Cosine similarity | HMDD V3.0, dbDEMC, miRCancer | Deep belief network  and matrix  factorization | Take full advantage of  data | The calculation Is based on  miRNA-disease associations. | NA |  |  |  |  |  |  |
| **Graph neural network methods** | | | | | | |  |  |  |  |  |  |
| VGAE-MDA[17] | DSSM,  MFSM and GIP  kernel similarity for diseases/miRNA | HMDD v2.0 | Variational graph  AutoEncoders and GCN | This method considers  both network features  and distribution features | The algorithm complexity is  relatively high which is not  friendly when the database  becomes really big | NA |  |  |  |  |  |  |
| PBMDA[18] | DSSM,  MFSM and GIP  kernel similarity for diseases/miRNA | HMDD v2.0 | special depth-first search algorithm  heterogeneous graph | Ability to extract potential features. | The sparsity of matrix will affect final results | http://www.escience.cn/system/file?fileId=84394^#^ |  |  |  |  |  |  |
| GraRep[19] | Disease semantic similarity | lncRNASNP2, miRTarBase,lncRNASNP, LncRNADisease, LncRNA2Target v2.0, DrugBank5.0, STRING, DisGeNET,HMDD v3.0  and miRbase | Network  embedding-based  heterogeneous  information  integration method | Efficient and simple to  implement | Sensitivity is not good. | https://github.com/look0012/iCDA-CGR^#^ |  |  |  |  |  |  |
| NIMCGCN[20] | HMDD v2.0/MiRNA function similarity, GIP  kernel similarity and semantic  similarity | dbDEMC v2.0, miR2Disease, miRCancer | Neural IMC with  GCN | High accuracy. | The structural information  affects final results. | https://github.com/ljatynu/NIMCGCN/ |  |  |  |  |  |  |

**Table S2** Summary of the corresponding MDAs’ information in the traning set and four different test sets obtained from HMDD v2.0 and HMDD v3.0

| **Datasets** | **Training set** | **Test set** | | | |
| --- | --- | --- | --- | --- | --- |
|  |  | **Tp** | **Td** | **Tm** | **Tn** |
| **Disease** | Old | Old | New | Old | New |
| **MiRNA** | Old | Old | Old | New | New |
| **Associations** | Old | New | New | New | New |

**Table S3** Detailed information about hyperparameters and architectures of ReHoGCN model (the same as UReHoGCN and HeGCN).

| **network** | dimensions | 512 |
| --- | --- | --- |
|  | aggregatation | concat |
|  | loss | sigmoid |
|  | architecture | four layers: GCN-SGCN-GCN-SGCN |
|  | activation | Relu |
|  | bias | norm |
| **parameters** | learning rate | 0.001 |
|  | dropout | 0.1 |
|  | weight decay | 0.0 |
|  | sample coverage | 50 |
|  | positive weight | 1 |
| **phase** | epoch | 1000 |
|  | sampler | edge |
|  | Number of subgraphs | 200 |
|  | Size of subgraph edges | 6000 |

**Figure S1** The effect of *k*-NN for ReHoGCNES model performance on training dataset via 10$\times$5 cross-validation, where the abscissa axis represents the self-loop (*k* =0) and the number of neighbors of each node (i.e. 1, 5, 10, 15).

When *k* equals 0, it establishes an edge from a node to the node itself, and each node in the graph has no neighbor nodes. Thus it doesn’t fully utilize the neighbor information resulting in unsatisfying performance of self-loop ReHoGCNES model. When *k* get larger, it can perform node updates, thus the AUC is higher. It also indicates that the proposed ReHoGCNES is not very sensitive to *k* and its robustness to the edge-building step is proven, which will avoid a lot of work in parameter tuning. Taking overall prediction performance and computation time into consideration, we choose best *k* is 5.

**Figure S2**. The effect of *k*-means for UReHoGCNES model performance on training dataset via 10$\times$5 cross-validation, where the abscissa axis represents the number of communities clustered by *k*-means (i.e. 100,200,300,400,500). Taking overall model performance and computation time into consideration, we choose best *k* is 300.

**Figure S3** Degree distribution of the three proposed models. A point $(k,p)$ in the plot means the probability of a node having degree at least $k$ is $p$.

**Table S4** Prediction performance of our proposed methods ReHoGCN compared with UReHoGCN and HeGCN with/without edge sampler on four tasks.

| **Task** | **Model** | **Accuracy** | **Precision** | **Recall** | **F1-Score** | **AUC** |
| --- | --- | --- | --- | --- | --- | --- |
| **Tp** | ReHoGCNES | 0.9957 | 0.9979 | 0.9936 | 0.9957 | 0.9998 |
|  | ReHoGCN | 0.9964 | 1.0000 | 0.9906 | 0.9953 | 0.9996 |
|  | UReHoGCNES | 0.9648 | 0.9682 | 0.9610 | 0.9646 | 0.9919 |
|  | UReHoGCN | 0.9645 | 0.9695 | 0.9603 | 0.9643 | 0.9923 |
|  | HeGCNES | 0.9279 | 0.9243 | 0.9277 | 0.9306 | 0.9645 |
|  | HeGCN | 0.9277 | 0.9240 | 0.9273 | 0.9312 | 0.9650 |
| **Td** | ReHoGCNES | 0.9799 | 0.9909 | 0.9688 | 0.9797 | 0.9982 |
|  | ReHoGCN | 0.9788 | 0.9912 | 0.9678 | 0.9798 | 0.9985 |
|  | UReHoGCNES | 0.9597 | 0.9537 | 0.9567 | 0.9502 | 0.9842 |
|  | UReHoGCN | 0.9588 | 0.9536 | 0.9559 | 0.9500 | 0.9839 |
|  | HeGCNES | 0.9287 | 0.9423 | 0.9299 | 0.9278 | 0.9511 |
|  | HeGCN | 0.9275 | 0.9430 | 0.9297 | 0.9273 | 0.9489 |
| **Tm** | ReHoGCNES | 0.9870 | 0.9931 | 0.9808 | 0.9869 | 0.9990 |
|  | ReHoGCN | 0.9865 | 0.9924 | 0.9823 | 0.9854 | 0.9985 |
|  | UReHoGCNES | 0.9411 | 0.9489 | 0.9327 | 0.9407 | 0.9816 |
|  | UReHoGCN | 0.9451 | 0.9404 | 0.9394 | 0.9448 | 0.9825 |
|  | HeGCNES | 0.9304 | 0.9346 | 0.9360 | 0.9303 | 0.9534 |
|  | HeGCN | 0.9322 | 0.9342 | 0.9379 | 0.9287 | 0.9540 |
| **Tn** | ReHoGCNES | 0.9581 | 0.9751 | 0.9402 | 0.9573 | 0.9944 |
|  | ReHoGCN | 0.9567 | 0.9742 | 0.9422 | 0.9570 | 0.9940 |
|  | UReHoGCNES | 0.9442 | 0.9484 | 0.9495 | 0.9440 | 0.9791 |
|  | UReHoGCN | 0.9427 | 0.9480 | 0.9489 | 0.9446 | 0.9734 |
|  | HeGCNES | 0.9247 | 0.9294 | 0.9296 | 0.9245 | 0.9414 |
|  | HeGCN | 0.9247 | 0.9286 | 0.9293 | 0.924 | 0.9372 |

**Table S5** Total running time (unit: sec) of our proposed methods ReHoGCN compared with UReHoGCN and HeGCN with/without edge sampler.

|  | **Tp** | **Tm** | **Td** | **Tn** |
| --- | --- | --- | --- | --- |
| **ReHoGCNES** | 590.42 | 522.14 | 614.71 | 279.51 |
| **ReHoGCN** | 3698.70 | 3874.35 | 2702.73 | 2342.57 |
| **UReHoGCNES** | 2273.73 | 1863.12 | 2595.17 | 1108.94 |
| **UReHoGCN** | 22770.30 | 20190.70 | 27720.58 | 12140.05 |
| **HeGCNES** | 880.33 | 803.54 | 764.43 | 326.74 |
| **HeGCN** | 974.85 | 932.77 | 867.32 | 389.67 |

**Table S6** Memory access cost (unit: MB) of our proposed methods ReHoGCN compared with UReHoGCN and HeGCN with/without edge sampler.

|  | **Tp** | **Tm** | **Td** | **Tn** |
| --- | --- | --- | --- | --- |
| **ReHoGCNES** | 2081 | 2109 | 2198 | 1023 |
| **ReHoGCN** | 4066 | 4273 | 4209 | 2548 |
| **UReHoGCNES** | 5273 | 5354 | 5328 | 3289 |
| **UReHoGCN** | 8235 | 8157 | 8392 | 4687 |
| **HeGCNES** | 1077 | 1234 | 1148 | 469 |
| **HeGCN** | 1225 | 1387 | 1399 | 542 |

**Table S7** P-value of prediction performance among three methods ReHoGCN, UReHoGCN and HeGCN with/without edge sampler on four MDA prediction tasks.

|  | **ReHoGCN** | **UReHoGCN** | **HeGCN** |
| --- | --- | --- | --- |
|  | ReHoGCNES | UReHoGCNES | HeGCNES |
| **Tp** | 0.938425 | 0.992249 | 0.997087 |
| **Tm** | 0.918331 | 0.958352 | 0.970546 |
| **Td** | 0.937899 | 0.905643 | 0.942078 |
| **Tn** | 0.987897 | 1 | 0.771993 |

**Table S8** P-value of prediction performance among three methods ReHoGCN, UReHoGCN and HeGCN with/without edge sampler on four MDA prediction tasks.

|  | **ReHoGCN** | **UReHoGCN** | **HeGCN** |
| --- | --- | --- | --- |
|  | ReHoGCNES | UReHoGCNES | HeGCNES |
| **Running time** | 0.00044 | 0.001224 | 0.616051 |
| **Memory cost** | 0.008231 | 0.04748 | 0.579886 |

P value: the probability of student t test; Type: double sample equal variance hypothesis; Tails: The two-tail distribution. Generally, P < 0.05 is significant and P <0.01 is very significant, meaning that the difference probability among samples due to sampling error is less than 0.05 or 0.01.

**Table S9** Average sampling time (unit: second) of our proposed methods ReHoGCN compared with UReHoGCN and HeGCN

| **Time (sec)** | **ReHoGCNES** | **UReHoGCNES** | **HeGCNES** |
| --- | --- | --- | --- |
| ReHoGCNES | 1.8 | 2.6 | 1.2 |

ReHoGCN+ES contains 54330 edges and UReHoGCN+ES contains 280605 edges while HeGCN contains 10860 edges (Tables 3 shows detailed degree statistics). We set number of subgraphs is 200 and size of subgraph edges is 6000 (see Table S3). That is, we use 200 subgraphs with 6000 edges to replace with full-graph training and we calculate total training time. Sampling time approximately equals 1.2 seconds (ten repeated sample, Table S6) for a HeGCNES graph and totally approximately takes 240 seconds. However, entire full-graph calculation of HeGCN model approximately takes 974.85 seconds (ten times repeated). The operation time of sampling probability calculation and subgraph generation are not negligible relative to the entire full-graph calculation. Full-graph training time of ReHoGCN and UReHoGCN model is much longer (3698.70s and 22770.30s) compared with sampling time (360s and 520s).

**Table S10** Results of ReHoGCNES performance compared with classic machine learning and deep learning models on four test sets obtained from HMDD.

| **Task** | **Methods** | **Accuracy** | **Precision** | **Recall** | **F1-Score** | **AUC** |
| --- | --- | --- | --- | --- | --- | --- |
| Tp | **ReHoGCNES** | **0.9957** | **0.9979** | **0.9936** | **0.9957** | **0.9998** |
|  | SVM | 0.5059 | 0.5038 | 0.9995 | 0.6699 | 0.5895 |
|  | RF | 0.8489 | 0.8470 | 0.8529 | 0.8499 | 0.9275 |
|  | GBDT | 0.8518 | 0.8452 | 0.8625 | 0.8538 | 0.9333 |
|  | DNN | 0.8456 | 0.8422 | 0.8519 | 0.8470 | 0.9267 |
| Td | **ReHoGCNES** | **0.9799** | **0.9909** | **0.9688** | **0.9797** | **0.9982** |
|  | SVM | 0.5062 | 0.5031 | 0.9993 | 0.6693 | 0.6172 |
|  | RF | 0.8055 | 0.8980 | 0.6893 | 0.7799 | 0.9098 |
|  | GBDT | 0.8108 | 0.9023 | 0.6971 | 0.7866 | 0.9190 |
|  | DNN | 0.7269 | 0.8788 | 0.5264 | 0.6584 | 0.7649 |
| Tm | **ReHoGCNES** | **0.9870** | **0.9931** | **0.9808** | **0.9869** | **0.9990** |
|  | SVM | 0.5068 | 0.5034 | 0.9993 | 0.6696 | 0.6071 |
|  | RF | 0.8403 | 0.9492 | 0.7191 | 0.8183 | 0.9505 |
|  | GBDT | 0.8417 | 0.9490 | 0.7223 | 0.8203 | 0.9531 |
|  | DNN | 0.7872 | 0.9473 | 0.6083 | 0.7409 | 0.9378 |
| Tn | **ReHoGCNES** | **0.9581** | **0.9751** | **0.9402** | **0.9573** | **0.9944** |
|  | SVM | 0.4993 | 0.4996 | 0.9884 | 0.6637 | 0.4881 |
|  | RF | 0.5022 | 0.5029 | 0.3759 | 0.4302 | 0.5026 |
|  | GBDT | 0.5065 | 0.5086 | 0.3861 | 0.4389 | 0.5047 |
|  | DNN | 0.5073 | 0.5110 | 0.3382 | 0.4070 | 0.5021 |

**Proof A**

**Lemma** *Let* $G=\left( V,E \right)$ *be a graph, and let* $0=\lambda_{1}\leq\lambda_{2}\leq\ldots\leq\lambda_{n}$ *be the eigenvalues of its Laplacian matrix. Then,* $\lambda_{2}>0$ *if and only if* $G$ *is connected.*

*Proof.* We first show that $\lambda_{2}=0$ if $G$ is connected. If $G$ is disconnected, then it can be described as the union of two graphs, $G_{1} \mathrm{and} G_{2}$*.* After suitably re-numbering the vertices, we can write

$L_{G}= \left[ \begin{matrix} L_{G1} & 0 \\ 0 & L_{G2} \end{matrix} \right]$*.*

So, $L_{G}$ has at least two orthogonal eigenvectors of eigenvalue zero:

$$\left[ \begin{matrix} 0 \\ 1 \end{matrix} \right]\mathrm{and}\left[ \begin{matrix} 1 \\ 0 \end{matrix} \right].$$

Where we have partitioned the vectors as we did the matrix $L_{G}$.

On the other hand, assume that $G$ is connected and that $x$ is an eigenvector of $L_{G}$ of eigenvalue 0.

$L_{G}x=0$*,*

We have

$$x^{T}L_{G}x= \sum_{(u,v)\in E} \left( x\left( u \right)-x\left( v \right) \right)^{2}=0$$

Thus, for each pair of vertices $(u,v)$ connected by an edge, we have $x\left( u \right)=x\left( v \right).$ As every pair of vertices $u$ and $v$ are connected by a path, we may inductively apply this fact to show that $x\left( u \right)=x(v)$ for all vertices $u \mathrm{and} v.$ Thus, $x$ must be a constant vector. We conclude that the eigenspace of eigenvalue 0 has dimension 1.

Of course, the same holds for weighted graphs.

GCN's Laplacian matrix eigenvalues have a very large relationship with the connectivity of the graph, and the second eigenvalue of the matrix is separately named the algebraic connectivity of the graph. Since the eigenvalues reflect the frequency characteristics of the graph structure, their value range has a great impact on the expression ability of GCN: that is, if all the eigenvalues are small, the value space of the frequency domain features will also be greatly limited. It is worth to note in the process of graph construction that there exists an $r$-regular graph of order $n$if and only if at least one of $r$ and $n$ is even.

References

1. Yan C, Wang J, Ni P et al. DNRLMF-MDA:Predicting microRNA-Disease Associations Based on Similarities of microRNAs and Diseases. *IEEE/ACM Trans Comput Biol Bioinform* 2019;16:233-243.

2. Chen X, Zhou Z, Zhao Y. ELLPMDA: Ensemble learning and link prediction for miRNA-disease association prediction. *Rna Biology* 2018;15:807-818.

3. Chen X, Yan CC, Zhang X et al. HGIMDA: Heterogeneous graph inference for miRNA-disease association prediction. *Oncotarget* 2016;7:65257-65269.

4. Chen M, Liao B, Li Z. Global Similarity Method Based on a Two-tier Random Walk for the Prediction of microRNA-Disease Association. *Scientific Reports* 2018;8.

5. Liang C, Yu S, Luo J. Adaptive multi-view multi-label learning for identifying disease-associated candidate miRNAs. *Plos Computational Biology* 2019;15.

6. Chen X, Wu Q-F, Yan G-Y. RKNNMDA: Ranking-based KNN for MiRNA-Disease Association prediction. *Rna Biology* 2017;14:952-962.

7. Chen X, Yan CC, Zhang X et al. RBMMMDA: predicting multiple types of disease-microRNA associations. *Scientific Reports* 2015;5.

8. Chen X, Huang L, Xie D et al. EGBMMDA: Extreme Gradient Boosting Machine for MiRNA-Disease Association prediction. *Cell Death & Disease* 2018;9.

24. Chen X, Wang C-C, Yin J et al. Novel Human miRNA-Disease Association Inference Based on Random Forest. *Molecular Therapy-Nucleic Acids* 2018;13:568-579.

9. Wang L, You Z-H, Chen X et al. LMTRDA: Using logistic model tree to predict MiRNA-disease associations by fusing multi-source information of sequences and similarities. *Plos Computational Biology* 2019;15.

10. Zhao Y, Chen X, Yin J. Adaptive boosting-based computational model for predicting potential miRNA-disease associations. *Bioinformatics* 2019;35:4730-4738.

11. Chen X, Jiang Z-C, Xie D et al. A novel computational model based on super-disease and miRNA for potential miRNA-disease association prediction. *Molecular* *Biosystems* 2017;13:1202-1212

12. Fu L, Peng Q. A deep ensemble model to predict miRNA-disease association. *Scientific Reports* 2017;7.

13. Fu L, Peng Q. A deep ensemble model to predict miRNA-disease association. *Scientific Reports* 2017;7.

14. Ji C, Gao Z, Ma X et al. AEMDA: inferring miRNA-disease associations based on deep autoencoder. *Bioinformatics* 2021;37:66-72.

15. Yu D-L, Ma Y-L, Yu Z-G. Inferring microRNA-disease association by hybrid recommendation algorithm and unbalanced bi-random walk on heterogeneous network. *Scientific Reports* 2019;9.

16. Ding Y, Wang F, Lei X et al. Deep belief network-Based Matrix Factorization Model for MicroRNA-Disease Associations Prediction. *Evolutionary Bioinformatics* 2020;16.

17. Ding Y, Tian L-P, Lei X et al. Variational graph auto-encoders for miRNA-disease association prediction. *Methods* 2021;192:25-34.

18. You Z-H, Huang Z-A, Zhu Z et al. PBMDA: A novel and effective path-based computational model for miRNA-disease association prediction. *Plos Computational Biology* 2017;13

19. Zheng K, You Z-H, Li J-Q et al. iCDA-CGR: Identification of circRNA-disease associations based on Chaos Game Representation. *Plos Computational Biology* 2020;16.

20. Li J, Zhang S, Liu T et al. Neural inductive matrix completion with graph convolutional networks for miRNA-disease association prediction. *Bioinformatics* 2020;36:2538-2546.
